# Editorial: The neurology and psychology of time perception

**DOI:** 10.3389/fneur.2023.1282635

**Published:** 2023-09-20

**Authors:** Deana Davalos, Edison K. Miyawaki

**Affiliations:** ^1^Department of Psychology, Colorado State University, Fort Collins, CO, United States; ^2^Department of Neurology, Mass General Brigham, Harvard Medical School, Boston, MA, United States

**Keywords:** time, perception, dopamine, three dimensional, rhythm, error, functional imaging, meta analysis

In everyday life, experience varies its tempo: time can pass quickly; sometimes, all too slowly. The five articles in our Research Topic, entitled *The neurology and psychology of time perception*, address novel ways to study such variance, and they introduce the reader to the still-growing study of perception as a function of time.

Miyawaki reviews human studies over decades that explore subjective time perception in relationship to dopamine neurotransmission in health and disease. He introduces basic concepts. An important distinction is that between explicit and implicit timing. An explicit timing task asks that a subject provide a specific estimation of a time interval. For example, in a time reproduction task, a subject is presented with a target interval visually or aurally, then, later, she is asked to perform some movement (e.g., sustained key press) for the amount of time that the target interval had been presented. By contrast, implicit timing does not require a specific estimation of how long a stimulus or action lasts. Rather, a subject *enlists* time either to accomplish a task or to predict when something will occur in relationship to a prior event. The gist of an implicit task relates to how time underpins action, as in the time it takes for nerve stimulation to induce contraction in a muscle that the nerve innervates.

An explicit task informs Ueda and Shimoda's study of how an action influences the reproduction of an auditory tone presented after the action. The authors argue that reproduced time intervals shorten independently of feedback stimuli delivered during the action, although the feedback influences a “sense of agency” experienced by performers of the action. As with many investigations into temporal perception, it can be hard to discern exactly what a study teaches us unless we think of an everyday analogy. If we drive a car, does feedback during the drive increase our “agency” (“indeed, I am driving the car”), and, importantly, does time after a drive seem shorter, having driven oneself? Does agency influence the perception of outcome? The question relates to the discerned effect of what we want—or will—to do in any act.

The chronobiology literature, which traverses anatomy from the hypothalamus (involved in the percept of circadian variation) to diverse other parts of the brain, assumes to some degree that time is a parameter independent of others. Yet, Yabe and Yamada examine how past, present, and future relate to each other both in terms of space and time. Leveraging a psychological test from the 1960s, the authors explore the judgment of earlier and later events based on how the subjects position events graphically in two dimensions or in three. Cultural differences emerge. Just in two dimensions, leftwardness indicates pastness for English-speaking persons; rightwardness and upwardness indicate pastness for those whose written language proceeds from right to left and from top to bottom, as in traditional Japanese. The third dimension introduces another nuance: time is a kind of space—e.g., “in front of,” in Japanese, indicates both earliness and “frontness”—either the temporal past or the face, say, of a 3-D structure.

Mansuri et al. explore an implicit timing mechanism involved in recognition of an ongoing rhythm or beat. Again, consider an analogy: in spoken English, there is rhythm, typically three to five syllables per second, or roughly 200-300 milliseconds between syllables. If the inter-syllable time is shorter (as occurs when a taped lecture is played at a faster speed), then the perceived time between a later syllable and the one that immediately precedes it is also shorter. If the inter-syllable interval is comparatively long overall (slower tape speed), the perceived time between a late syllable and the preceding one is also longer. The authors mathematically analyze data obtained in epochs of largely (but not perfectly) fixed rhythms. Perceptual errors of the two types just described occur, and error (either shorter or longer) lessens if a subject attends to a rhythm for a longer epoch. But absolute correction does not occur. The authors argue that such seemingly systematic misperception may serve a purpose, because biological timing is not meant to measure perfectly, but rather to be *useful in context*, as when danger is imminent in a changing environment.

Mondok and Wiener address an intricately complex topic: which areas of the brain allow us to sense the passage of time? They interrogated a large number of functional imaging studies that examined explicit timing tasks and other reports that specifically did not employ explicit tasks (studies that did not examine time perception at all), then undertook a kind of reverse analysis. They did not direct their attention to brain areas that activate in an explicit timing task. Instead, using a respected, if still-debated meta-analytic tool (Bayes factor modeling), they asked: if a brain area activates, what is the likelihood (what is the posterior probability) that the subject was involved in a timing task? They concluded that the left supplementary motor area in the medial frontal lobe and bilateral insula exhibited the highest posterior probabilities of activation in explicit timing tasks.

As with all the studies in our small Research Topic, interest is greatest when one considers questions about time that arise from the reports. Why have human investigations into time perception been so diverse in their findings? What change in temporal perception happens after an action? Does one, should one, refer to time-space? Is rhythm specifically not like a metronome? Does posterior probability of activation invert how we should think about functional brain imaging generally?

The editors thank the reviewers for their very considered comments and especially the authors, who undertook to answer all criticisms to the best of their ability.

## Author contributions

DD: Writing–original draft, Writing–review and editing. EM: Writing–original draft, Writing–review and editing.

